# Tubulin perturbation leads to unexpected cell wall modifications and affects stomatal behaviour in *Populus*

**DOI:** 10.1093/jxb/erv383

**Published:** 2015-08-05

**Authors:** Prashant S. Swamy, Hao Hu, Sivakumar Pattathil, Victoria J. Maloney, Hui Xiao, Liang-Jiao Xue, Jeng-Der Chung, Virgil E. Johnson, Yingying Zhu, Gary F. Peter, Michael G. Hahn, Shawn D. Mansfield, Scott A. Harding, Chung-Jui Tsai

**Affiliations:** ^1^School of Forestry and Natural Resources, University of Georgia, Athens, GA 30602, USA; ^2^Department of Genetics, University of Georgia, Athens, GA 30602, USA; ^3^Complex Carbohydrate Research Center, University of Georgia, Athens, GA 30602, USA; ^4^Department of Wood Science, University of British Columbia, Vancouver, BC V6T 1Z4, Canada; ^5^Laboratory for Macromolecular Analysis and Proteomics, Albert Einstein College of Medicine, Bronx, NY 10461, USA; ^6^Division of Silviculture, Taiwan Forestry Research Institute, Taipei 10066, Taiwan; ^7^School of Forest Resources and Conservation, University of Florida, Gainesville, FL 32611, USA

**Keywords:** Cell wall, detyrosination, guard cell, microtubule, polysaccharide, tubulin post translational modification.

## Abstract

Cortical microtubules are integral to plant morphogenesis, cell wall synthesis, and stomatal behaviour, presumably by governing cellulose microfibril orientation. Genetic manipulation of tubulins often leads to abnormal plant development, making it difficult to probe additional roles of cortical microtubules in cell wall biogenesis. Here, it is shown that expressing post-translational C-terminal modification mimics of α-tubulin altered cell wall characteristics and guard cell dynamics in transgenic *Populus tremula* x *alba* that otherwise appear normal. 35S promoter-driven transgene expression was high in leaves but unusually low in xylem, suggesting high levels of tubulin transgene expression were not tolerated in wood-forming tissues during regeneration of transformants. Cellulose, hemicellulose, and lignin contents were unaffected in transgenic wood, but expression of cell wall-modifying enzymes, and extractability of lignin-bound pectin and xylan polysaccharides were increased in developing xylem. The results suggest that pectin and xylan polysaccharides deposited early during cell wall biogenesis are more sensitive to subtle tubulin perturbation than cellulose and matrix polysaccharides deposited later. Tubulin perturbation also affected guard cell behaviour, delaying drought-induced stomatal closure as well as light-induced stomatal opening in leaves. Pectins have been shown to confer cell wall flexibility critical for reversible stomatal movement, and results presented here are consistent with microtubule involvement in this process. Taken together, the data show the value of growth-compatible tubulin perturbations for discerning microtubule functions, and add to the growing body of evidence for microtubule involvement in non-cellulosic polysaccharide assembly during cell wall biogenesis.

## Highlight

Growth-compatible, post-translational modifications of tubulin in transgenic Populus alter cell wall pectin-xylan networks and guard cell movement.

## Introduction

In plants, cortical microtubules (MTs) play essential roles in cell expansion, cell wall biogenesis, and stomatal behaviour via their influence over cellulose microfibril (MF) deposition (Ledbetter and Porter, 1963; Wasteneys, 2002; Paredez *et al.*, 2006; Eisinger *et al.*, 2012). It is now established that this classic function depends on MT-mediated membrane trafficking of the cellulose synthase complexes (Crowell *et al.*, 2009; Gutierrez *et al.*, 2009). MTs are also intimately linked to organelle trafficking (Crowell *et al.*, 2009; Hamada *et al.*, 2012), and have been recently implicated in intracellular movement of cell wall matrix components to the cell cortex (Kong *et al.*, 2015; Zhu *et al.*, 2015). Consistent with this role, MTs aggregate near sites of pectin mucilage secretion in the seed coat of *Arabidopsis* (McFarlane *et al.*, 2008). Disruption of the MT–MF alignment by cobtorin in tobacco BY-2 cells altered pectin distribution, which implicated not only pectins in MT–MF alignment, but also MTs in pectin deposition (Yoneda *et al.*, 2010). Similarly, depolymerization of MTs by oryzalin affected not only cellulose but also pectin deposition in *Penium margaritaceum* (Domozych *et al.*, 2014).

Genetic manipulation of MTs for functional investigation is challenging, owing not only to the multiplicity of MT functions, but also to the complex transcriptional and post-translational regulation of the tubulin subunits. MT function depends on the polymerization and depolymerization of α-tubulin (TUA) and β-tubulin (TUB) heterodimers in response to developmental and environmental cues (Ehrhardt and Shaw, 2006). TUA and TUB are encoded by multi-gene families in all sequenced plant genomes, with the woody perennial *Populus* harbouring eight *TUA* and 20 *TUB* genes (Oakley *et al.*, 2007). Biased expansion of the *Populus TUB* family coincides with greatly reduced transcript levels of individual genes when compared to the *TUA* family, implicating a complex *TUA*:*TUB* transcriptional regulation (Oakley *et al.*, 2007). Interestingly, overexpression of *TUB*, but not *TUA*, led to rapid loss of viability in yeast (*Saccharomyces cerevisiae*), suggesting that TUB in excess of TUA is lethal (Burke *et al.*, 1989; Weinstein and Solomon, 1990). In tobacco, co-transformation of *TUA* and *TUB* was deemed necessary in order to obtain viable transformants (Anthony *et al.*, 1999). Despite the potential challenges, it has been possible to recover *Arabidopsis* with defective tubulin synthesis. However, many such plants display abnormal organ development, cell wall synthesis, and growth (Burk *et al.*, 2006; Ishida *et al.*, 2007), making it difficult to discern direct and indirect effects of MT perturbations.

In animals, tubulins undergo extensive post-translational modifications (PTMs) that affect MT dynamics (Janke and Bulinski, 2011). One such modification involves the evolutionarily conserved C-terminal Tyr of TUA, which can be enzymatically removed or re-attached via the tyrosination–detyrosination cycle (Janke and Bulinski, 2011). The detyrosinated (dY) TUA may be irreversibly converted to non-tyrosinatable (Δ2 or dEY) TUA by removal of the penultimate Glu (Paturle-Lafanechere *et al.*, 1991). These PTM isoforms exhibit spatiotemporal distribution and are enriched in long-lived stable MTs during mammalian development (Gundersen *et al.*, 1984; Webster *et al.*, 1987; Peris *et al.*, 2009). In contrast, tubulin tyrosination–detyrosination is poorly understood in plants. Putative dY and dEY TUA isoforms have been reported in plants using animal tubulin PTM-specific antibodies (Duckett and Lloyd, 1994; Smertenko *et al.*, 1997; Wang *et al.*, 2004), but evidence by direct proteomics analysis is lacking.

The current investigation exploited tubulin PTMs to perturb MT regulation and dynamics in *Populus tremula* x *alba* 717-1B4. Expression of dY or dEY PTM mimics of TUA1 was found to facilitate recovery of transgenic *Populus* that was otherwise unsuccessful by co-transformation with native *TUA* and *TUB* genes. This permitted an analysis of multiple MT-dependent processes in transgenic *Populus*. Perturbation of tubulin PTMs was found to affect cell wall pectin–xylan organization, alter expression of cell wall-modifying enzymes, and attenuate transcriptional response to gravitational stress in wood-forming tissues. In addition, stomatal responses to environmental cues were affected in transgenic leaves. These findings that non-cellulosic carbohydrate organization and guard cell behaviour are sensitive to tubulin PTMs support the emerging role of MTs in matrix polysaccharide network assembly.

## Materials and methods

### Generation of transgenic poplars and quantitative RT-PCR

Coding sequences of *TUA1* (or its 3′-truncated PTM mimics), *TUA5*, *TUB9*, or *TUB15* were PCR-amplified from *P. tremula* × *alba* 717-1B4 xylem cDNA, cloned into pCR2.0-TOPO (Invitrogen), and sequence-confirmed. The *Xba* I-*Sma* I *TUA* fragments were subcloned into binary vector pCambia1302 at *Spe* I and *Pml* I, with the hygromycin selectable marker. *TUB* genes were similarly cloned into a modified pCambia2301 (with its *Eco* RI-*Pml* I fragment replaced with that of pCambia1302), with the kanamycin selectable marker. After mobilization into *Agrobacterium tumefaciens* C58/pMP90, *TUA* and *TUB* constructs were mixed in equal ratio for co-transformation into *P. tremula* × *alba* 717-1B4 (Meilan and Ma, 2006). Putative transformants were selected on media containing both kanamycin and hygromycin, and PCR-confirmed. Plant propagation and greenhouse maintenance were as described (Frost *et al.*, 2012). Leaf plastochron index (LPI)-15 and developing xylem from internodes 60–80 were snap-frozen in liquid N for RNA extraction. Internodes 50–60 were debarked and air-dried for wood chemistry analysis. Total RNA extraction for real-time qRT-PCR analysis was performed as described (Oakley *et al.*, 2007). A vector-specific primer was used in conjunction with *TUA*/*TUB*-specific primers to differentiate between transgenes and their endogenous counterparts (see Supplementary Table S1 for primers).

### Histology

Paraffin stem sections were prepared as described (Tsai and Harding, 2013), stained with 0.05% (w/v) toluidine blue and imaged with a Zeiss Axioskop-50 microscope.

### Tubulin proteomics and western blot analysis

Tubulin was purified from developing xylem (10g) by DEAE-Sephadex chromatography as described (Smertenko *et al.*, 1997), with a modified buffer-to-tissue ratio of two. Tubulin-enriched fractions were dialyzed, concentrated using a Nanosep centrifugal column (MWCO 10K, Pall Life Sciences), and an aliquot (~5 µg) separated by 10% SDS-PAGE. Bands corresponding to tubulins (~50kDa) were excised and subjected to in-gel cyanogen bromide and trypsin digestion for proteomics analysis by LC-matrix-assisted laser desorption/ionization (MALDI) and MALDI time-of-flight tandem (TOF-TOF) mass spectrometry as described (Xiao *et al.*, 2009) at the Proteomics core, Albert Einstein College of Medicine. Relative abundance of TUA1 and its PTM variants was estimated using the label-free method (Miller *et al.*, 2012). For western blot analysis, crude proteins were extracted from leaves and xylem as described (Deng *et al.*, 2007). Five micrograms of proteins were resolved by 10% SDS-PAGE and electro-transferred onto Immobilon-FL PVDF membrane (EMD Millipore). Replicate blots were hybridized with polyclonal antibodies raised in rabbits against affinity-purified recombinant TUA1 at 1:5000 dilution (anti-TUA1, Open Biosystems) or synthetic C-terminal peptides of TUA1 at 1:10 000 dilution (anti-dY: ESPDGEDGDEGDE or anti-dEY: ESPDGEDGDEGD, Sigma Genosys). Hybridization signals were detected using IRDye 680RD-conjugated goat anti-rabbit IgG secondary antibodies and an Odyssey infrared imaging system (Licor).

### Wood chemical and physical properties, and tension wood induction

Stem wood was Wiley-milled (40-mesh), Soxhlet-extracted, and air-dried. Lignin content was determined by the Klason method and an aliquot of the acid hydrolysate was used for structural carbohydrate analysis by anion exchange HPLC (Porth *et al.*, 2013). Syringyl-to-guaiacyl (S/G) lignin ratio was determined by pyrolysis GC-MS (del Río *et al.*, 2001). Intact stem segments (~2cm) were used for wood density, microfibril angle (MFA) and cellulose crystallinity analyses (Porth *et al.*, 2013). A separate cohort of vegetatively propagated plants (~1 m height) was inclined at a 30° angle to induce tension wood (TW). After 3 weeks, the stem was debarked and developing xylem from the TW side of the basal half was collected for RNA extraction, while the distal half was air-dried and split lengthwise to obtain TW-enriched wood for chemical analysis as above. Stems from control, erect trees were processed identically. Intact stem segments (~1cm in diameter) were used for X-ray micro computed tomography (μCT) analysis of wood density as described (Joshi *et al.*, 2011).

### Glycome profiling and glycosyl composition analysis of cell wall extracts

Sequential cell wall extractions and glycome profiling of extractive-free wood meal were performed as described (DeMartini *et al.*, 2011; Pattathil *et al.*, 2012). Plant glycan-directed monoclonal antibodies (mAbs) were from CarboSource Services (CCRC, JIM and MAC series) at the Complex Carbohydrate Research Center, or BioSupplies (Australia) (BG1, LAMP). The data were filtered to remove weakly reacting mAbs (hybridization intensities <0.1 in all samples). The signal intensities from 127 mAbs were subjected to Z-score transformation (Cheadle *et al.*, 2003) and self-organizing map clustering using MeV v4.5 (Saeed *et al.*, 2003) and the bubble neighbourhood method (radius = 0.8). Plant samples within each group [wild type (WT), A1dYB9 or A1dEYB15+A1dEY] were pooled for statistical testing of transgenic effects using Limma (Smyth, 2005). The sequential cell wall extracts (200–500 μg per sample) were used for glycosyl composition analysis by GC-MS as described (York *et al.*, 1986; Merkle and Poppe, 1994) at the Complex Carbohydrate Research Center’s Analytical Services, University of Georgia.

### RNA-Seq

Total RNA was extracted using the Direct-zol kit (Zymo Research) with Plant RNA Reagent (Life Technologies). Illumina TruSeq RNA library preparation and HiSeq-2000 sequencing were performed at the Genomics Core, University of Texas Southwestern Medical Center. Eight to eighteen million paired-end 100-bp reads were generated per sample for four plant lines (WT, A1dYB9, A1dEYB15-11, and A1dEY-5), each with two to eight biological replicates. After filtering to remove organellar contaminants, sequences were mapped to the *Populus* genome v3.0 (Phytozome) and processed using the Tuxedo tools (Trapnell *et al.*, 2012). Differential expression was assessed for 9244 genes (FPKM ≥5 in all samples) to gauge transgenic or TW effects using Limma (Smyth, 2005) with multiple-testing corrections by SLIM (Wang *et al.*, 2011). The RNA-Seq data are available under NCBI Sequence Read Archive accession number SRP042117.

### Drought treatments and stomatal conductance

Vegetatively propagated plants of 1–1.5 m in height were used. Stomatal conductance was monitored on the same plants before and during the onset of drought response, or on plants maintained under different watering regimes. For drought treatment, water was withheld until the first visible sign of wilt in young leaves (typically 16–20h), at which point gas exchange, stomatal conductance, and transpiration rates were measured on LPI-15 using a Licor LI-6400XS as described (Frost *et al.*, 2012). Stomatal responses to light were measured at dawn. Data were analysed by paired two-sample *t* test or repeated measures ANOVA followed by the Student–Newman–Keuls post-hoc test using SigmaPlot 12 (Systat Software Inc.).

## Results

### Tubulin manipulation in transgenic *Populus* was facilitated by PTM mimics

Two xylem-abundant *TUA* (*TUA1*, *TUA5*) and *TUB* (*TUB9* and *TUB15*) genes (Oakley *et al.*, 2007) were selected for co-transformation into *Populus* in eight construct combinations: four as native gene pairs (referred to as A1B9, A1B15, A5B9, and A5B15) and four pairs with dY/dEY PTM mimics of *TUA1* in place of native *TUA1* (denoted as A1dYB9, A1dYB15, A1dEYB9, A1dEYB15). (Note, TUA5 carries a C-terminal methionine and is not predicted to participate in the tyrosination-detyrosination cycle (Oakley *et al.*, 2007)). The 35S promoter was used in all cases. Putative transgenic calli were obtained from all construct combinations, but the transformation efficiency was very low compared to the vector control in multiple trials. All of the callus lines derived from the native *TUA–TUB* construct pairs showed abnormal vascular development during organogenesis and eventually died (Supplementary Table S2, Supplementary Fig. S1). In contrast, the four pairs that contained PTM mimics had higher transformation efficiencies at the callus stage, and three produced viable transgenic plants (Supplemental Table S2). It appears that the *TUA* and *TUB* co-transformation strategy was not as effective in *Populus* as in tobacco (Anthony *et al.*, 1999) for regeneration of viable plants, and that the PTM mimic-containing tubulin gene pairs facilitated whole plant regeneration in *Populus*.

**Fig. 1.**
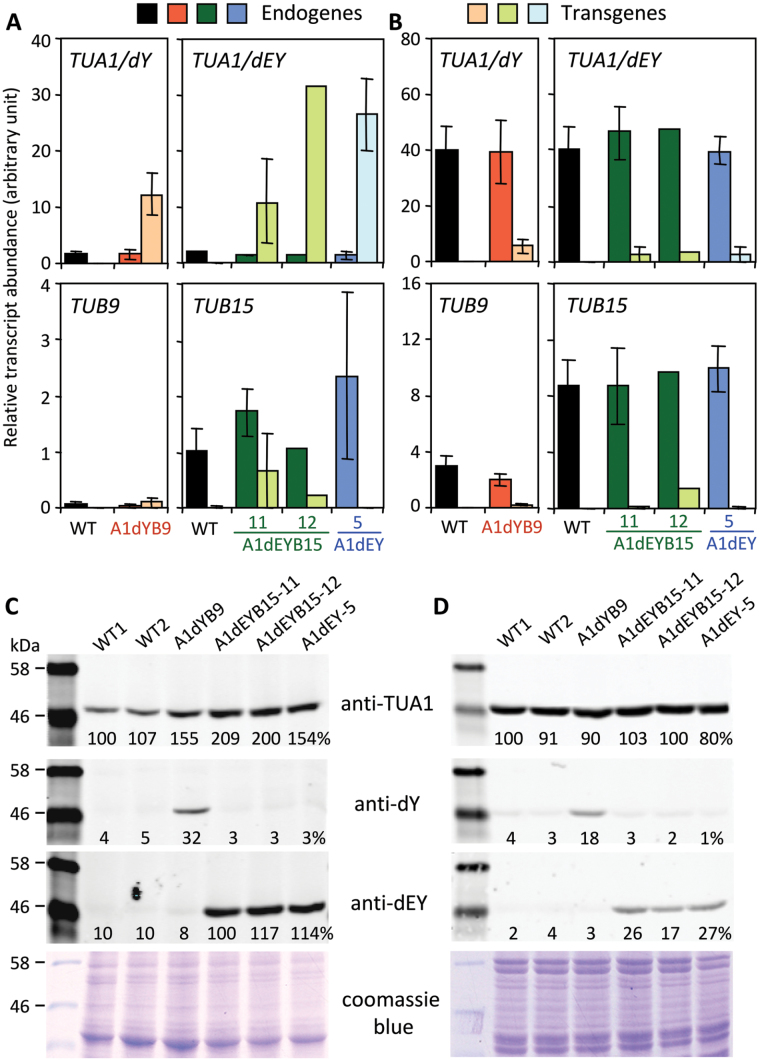
Tubulin transcript and protein accumulation. (A, B) qRT-PCR analysis of relative tubulin transcript abundance in leaves (A) and xylem (B). Data are mean ± SD of n = 3–7 biological replicates, except for A1dEY-5 (mean ± range of n = 2) and A1dEYB15-12 (n = 1). (C, D) Western blot analysis of TUA levels in leaves (C) and xylem (D) using polyclonal antibodies raised against recombinant TUA1 (anti-TUA1) or synthetic C-terminal peptides of TUA1 (anti-dY and anti-dEY). Relative (%) signal abundance normalized to marker bands was estimated within the anti-TUA1 blot for genotypic comparison, or across the three blots (anti-TUA1, anti-dY, and anti-dEY) for isoform comparison.

PCR analysis of genomic DNA confirmed the presence of the *TUA* and *TUB* transgenes in the transgenic lines, except for the presumed A1dEYB15-5 and A1dEYB15-17 lines which lacked the *TUB15* and *NPTII* PCR amplicons and were renamed A1dEY-5 and A1dEY-17 (Supplementary Fig. S2). The first cohort of transformants, including A1dYB9 (one line), A1dEYB15 (two lines), and A1dEY (one line) plants, was fully characterized. Endogenous transcript abundance was not changed in any of the transgenic lines (Fig. 1). The transcript abundance of *TUA1dY* or *TUA1dEY* in mature leaves (LPI-15) was 6–17-fold higher than endogenous *TUA1* in all transgenic lines (Fig. 1A). In contrast, expression of the *TUB* transgenes was low, and sometimes below endogene levels (Fig. 1A). In developing xylem where endogenous tubulin transcripts were abundant, the transgenes were detected at very low levels in all viable transgenic lines we obtained. This strongly suggested that high levels of tubulin transgene expression were not tolerated in xylem during organogenesis. As in leaves, the relative transcript abundance of *TUB* transgenes was lower than that of *TUA* transgenes (Fig. 1B). The results suggest that the *TUA* and *TUB* transgenes were, much like their endogenous counterparts, differentially regulated in a gene family- and tissue-dependent manner.

**Fig. 2.**
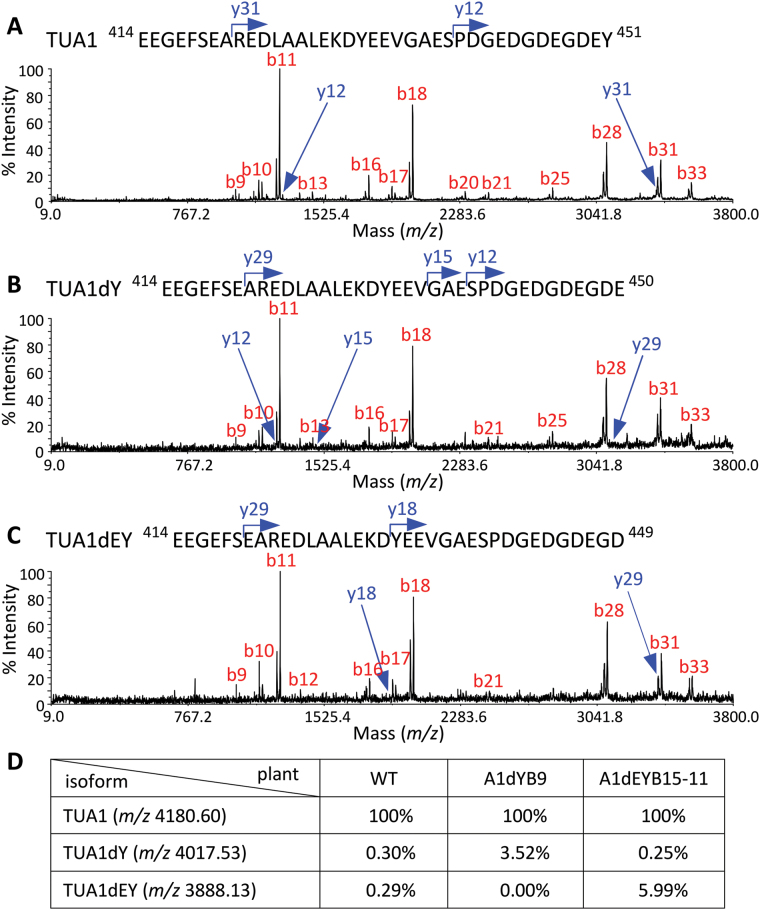
Proteomic analysis of tubulin-enriched xylem extracts. (A-C) MALDI TOF-TOF spectra of cyanogen bromide-cleaved C-terminal peptide of TUA1 (A), TUA1dY (B), and TUA1dEY (C) from WT, A1dYB9, and A1dEYB15 plants, respectively. (D) Relative abundance of the three TUA1 isoforms as determined by MALDI-TOF based on the C-terminal peptides. The peptide intensity of TUA1 was set to 100% in each sample.

The tissue-biased transgene expression was also detected at the protein level by western blot analysis. The total TUA (anti-TUA1) signal increased by as much as two-fold in transgenic leaves relative to the WT (Fig. 1C), but remained similar in xylem across genotypes (Fig. 1D). Anti-dY and anti-dEY signals were essentially absent in WT, but were readily detected in the respective transgenics. Specifically, anti-dEY signal was as strong as that of total TUA in leaves of dEYB15 and dEY plants, but accounted for only 17–27% of the total TUA signal in xylem of these plants (Fig. 1C,D). Proteomics analysis confirmed that the PTM mimics TUA1dY and/or TUA1dEY were present in transgenic xylem extracts (Fig. 2). Consistent with the western blot results, levels of the PTM isoforms were negligible in WT, but increased considerably in the transgenics, accounting for 3–6% of the TUA1 levels (Fig. 2D). In both western and proteomics analyses, the dY and dEY isoforms were only detected in the A1dYB9 and A1dEYB15-11 lines, respectively. This suggests that the enzymatic dY-to-dEY conversion reported in animal systems (Janke and Bulinski, 2011) may be absent in *Populus*. While the abundance estimate varied between detection platforms and cannot be directly compared, the results nevertheless provide protein-level evidence for active synthesis of TUA1 PTM mimics in the transgenics.

### Wood physicochemical properties were not quantitatively changed but pectin and xylan extractability was altered in the transgenics

Young stem cross sections of WT and transgenic plants were anatomically similar (Supplementary Fig. S3). Wood density, MFA, and cellulose crystallinity of the woody stems did not show transgenic effects (Table 1). Cell wall carbohydrate and lignin contents also did not differ between genotypes; however, the S/G monolignol ratio was lower in the transgenics (Table 1). The results suggested that tubulin perturbation in this system led to subtle compositional modifications, but not quantitative changes in major constituents of the cell wall.

**Table 1.** Wood characteristics

	Wood density	MFA	Crystallinity	Glucose	Xylose	Arabinose	Rhamnose	Galactose	Mannose	Lignin	S/G ratio
	Kg m-^3^	degree	%	%	%	%	%	%	%	%	
WT	413.62±45.27	35.26±7.50	43.05±1.22	43.20±0.53	18.82±0.30	0.36±0.05	0.58±0.03	0.87±0.07	1.49±0.03	21.37±0.49	2.23±0.16
A1dYB9	399.38±40.62	43.60±7.89	42.94±2.19	42.71±0.85	18.21±0.62	0.35±0.02	0.59±0.03	1.01±0.16	1.57±0.10	21.93±0.69	1.81±0.06**
A1dEYB15-11	371.39±61.25	42.00±9.71	43.59±1.71	42.00±0.46	18.64±0.35	0.33±0.03	0.56±0.01	0.78±0.08	1.41±0.02 *	21.12±0.71	1.93±0.09 *
A1dEYB15-12	423.23	51.27	46.96	42.98	18.76	0.32	0.55	0.83	1.49	20.74	1.97
A1dEY-5	404.49±48.01	47.55±5.93	45.42±3.35	43.34±0.46	18.36±0.74	0.31±0.02	0.56±0.02	0.82±0.02	1.64±0.05	21.29±0.14	2.14±0.01

Data are mean ± SD of n = 3–9, except A1dYB15-12 (n = 1) and A1dEY-5 (n = 2). Statistical significance was determined using the two-sample t-test (**P < 0.01; *P < 0.05).

**Fig. 3.**
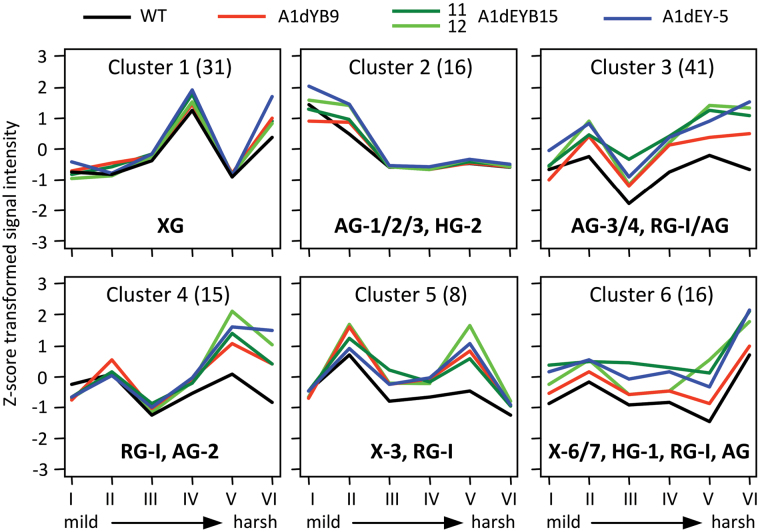
Glycome profiling of xylem. Self-organizing map clustering of 127 cell wall glycan epitopes in six sequential extracts of WT and transgenic xylem samples. The number of epitopes (in parentheses) and the predominant glycan class(es) in each cluster are indicated. The average signal intensities (Supplementary Table S3) are plotted against cell wall fractions and colour-coded by plant line for visualization. I-VI, sequential cell wall extractions with ammonium oxalate (I), sodium carbonate (II), 1M KOH (III), 4M KOH (IV), sodium chloride (V) and post-chlorite 4M KOH (VI). AG, arabinogalactan; HG, homogalacturonan; RG-I, rhamnogalacturonan I; X, xylan; XG, xyloglucan.

To further investigate possible cell wall modifications in the transgenics, extractive-free wood meal was subjected to sequential fractionation of cell wall polysaccharides for glycome profiling (Pattathil *et al.*, 2010; Pattathil *et al.*, 2012). In general, mild solvent extractions by oxalate (fraction I) and carbonate (II) preferentially release arabinogalactans (AGs) and pectic components. Subsequent alkaline extractions with 1M (III) and 4M KOH (IV) remove hemicellulosic components (xylans and xyloglucans). Chlorite extraction (V) degrades lignin and associated carbohydrates, with the post-chlorite 4M KOH treatment (VI) releasing additional tightly bound polysaccharides. Total sugars of each cell wall fraction estimated by the phenol-sulfuric acid method differed little between genotypes (Supplementary Fig. S4A). Self-organizing map clustering of ELISA data from 127 cell wall glycan-directed mAbs revealed six major epitope clusters across the cell wall fractions and genotypes (Fig. 3, Supplementary Table S3). Cluster 1 consisted mainly of xyloglucan epitopes, with similar extractability between genotypes (Fig. 3). The other clusters were dominated by pectin- (clusters 2–5) and/or xylan-derived (clusters 5 and 6) epitopes, and showed a general trend of increased extractability in the transgenics (Fig. 3). Statistical analysis confirmed that the vast majority of epitopes with significant changes (*P* ≤0.05 and fold-change ≥1.5) in both transgenic groups (39 out of 44) were derived from pectins (Supplementary Table S3).

The five pectin/xylan clusters revealed changes in the epitope extractability across cell wall fractions due to tubulin perturbation. Cluster 2 consisted of more easily-extracted pectic epitopes, such as AGs [classified under mAb groups AG-1 and AG-3 (Pattathil *et al.*, 2010)], arabinans (AG-2), and methyl-esterified homogalacturonans (HG-2). They were abundant in fractions I and II and showed modest transgenic effects (Fig. 3). In contrast, pectic epitopes in cluster 3 [AG-3 and rhamnogalacturonan I (RG-I/AG and AG-4)] and cluster 4 (RG-I backbone, RG-I/AG, arabinan and galactan) were present in all cell wall fractions and showed large transgenic effects (Fig. 3, Supplementary Table S3). Their extractability was most strongly enhanced in the transgenics after removal of lignin (fractions V and VI). The small cluster 5 contained both pectic (RG-I) and xylan (Xylan-3) epitopes with increased extractability in fractions II–V (Fig. 3). Cluster 6 included mostly xylan (Xylan-6 and Xylan-7) and a few pectic (RG-I/AG and de-esterified HG-1) epitopes that were abundant in all cell wall extracts, especially the most tightly bound fraction VI. Their extractability was more strongly affected in the A1dEYB15/A1dEY plants than the A1dYB9 line.

Glycosyl composition analysis of the four major cell wall fractions (III–VI) confirmed the glycome profiling results. Significantly higher levels of sugars were recovered from the A1dEY extracts (Supplementary Fig. S4B). Xyl was the predominant monosaccharide in fractions III, IV, and VI, while GalA was enriched in fraction V (Supplementary Fig. S4C-F). Xyl and Rha were recovered at significantly higher levels in fractions III, IV, and VI of A1dEYB15/A1dEY plants than in WT (Supplementary Fig. S4G,H,J). Lignin removal by chlorite (fraction V) released proportionately more non-Xyl monosaccharides, with GalA, Ara, Rha, and Gal higher in A1dEYB15/A1dEY than in WT plants (Supplementary Fig. S4I). The response of A1dYB9 was weaker. These results are interpreted to suggest that pectins and xylans deposited early during cell wall biogenesis are tightly associated with lignin, and most sensitive to tubulin perturbation. In contrast, subsequently deposited pectins that were more readily extracted by mild solvents were not as significantly affected in the transgenics. Thus, while cellulose and hemicelluloses were not quantitatively changed by the subtle tubulin manipulation reported here, the pectin–xylan polysaccharide organization, and their interactions with lignin polymers, appear to be modified in the transgenics.

### Elevated expression of cell wall-modifying enzymes and attenuated TW response in transgenic xylem

**Fig. 4.**
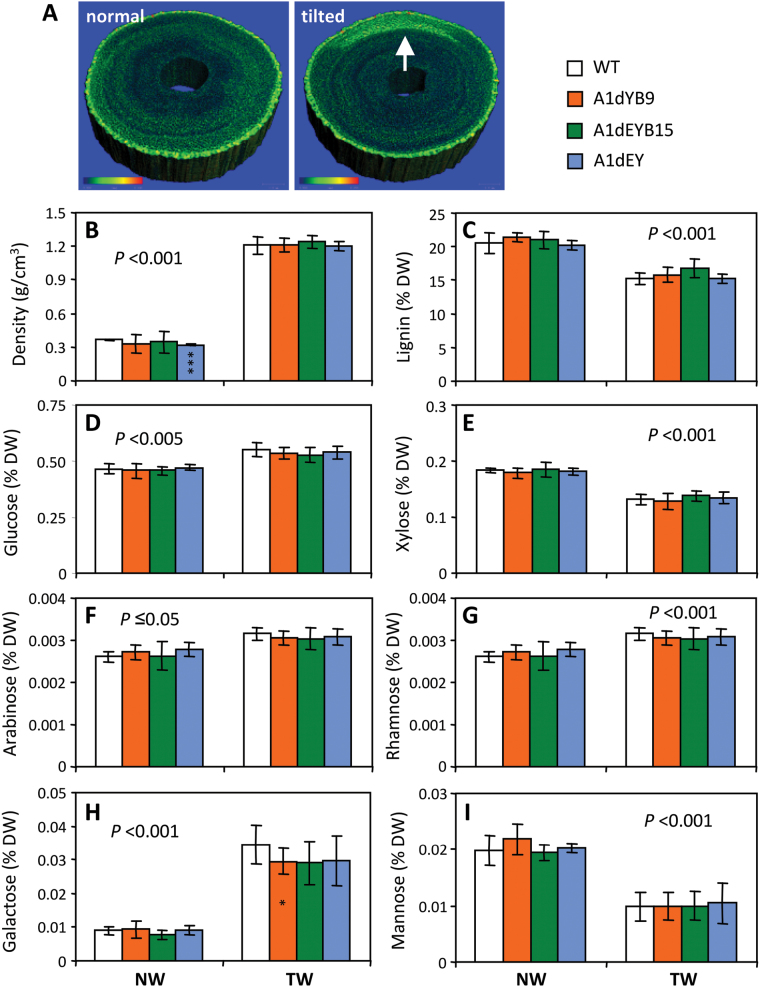
Characterization of tension wood. (A) Representative X-ray µCT images of normal and tilted stems. Bright pixels highlight the TW region (arrow) with increased cellulose deposition and wood density. (B) Wood density. (C) Lignin. (D-I) Structural carbohydrates including glucose (D), xylose (E), arabinose (F), rhamnose (G), galactose (H), and mannose (I). Error bars are SD of n = 4–10 plants. Statistical significance was determined using the two-sample *t* test. The *P* value in each panel denotes treatment effect (NW vs. TW) for all genotypes, while asterisks inside the bars indicate transgenic effect. ***P < 0.001; *P < 0.05.

**Fig. 5.**
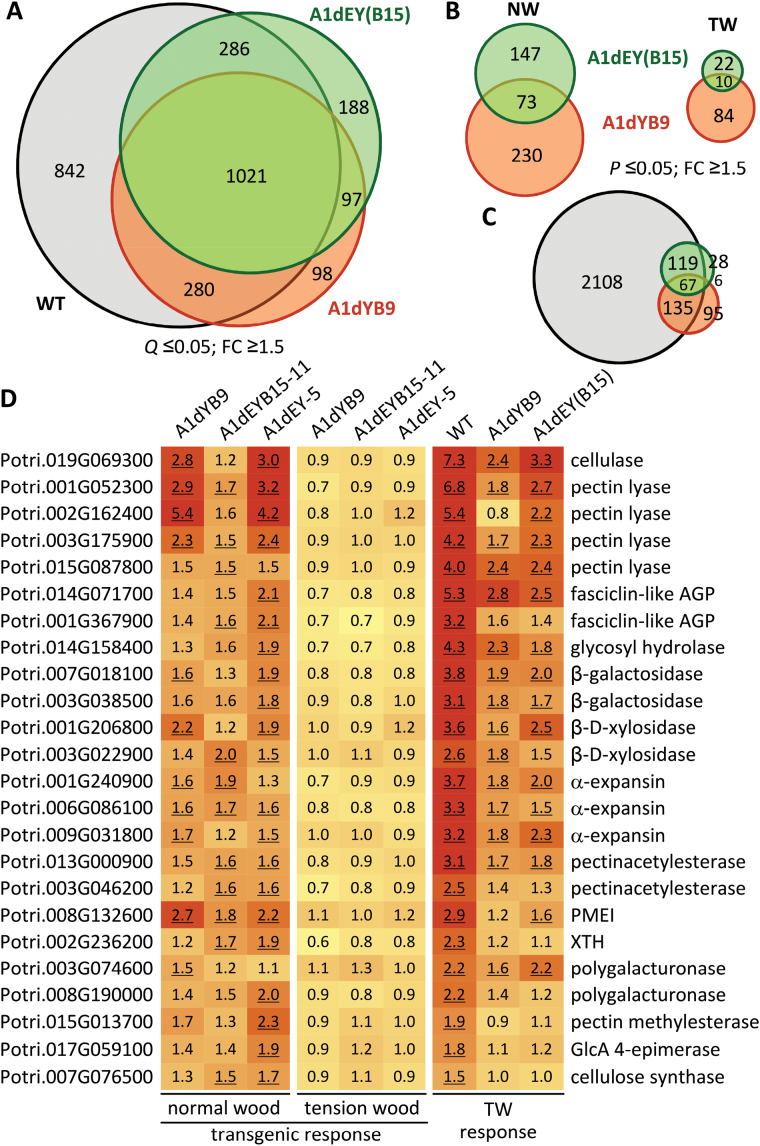
Gene expression analysis. (A) Venn diagram of genes with significantly altered expression in TW compared to NW, colour-coded by genotype. (B) Venn diagrams of genes with significant transgenic effects in NW and TW. (C) Venn diagram of genes significantly affected by TW in WT (from A) or by tubulin perturbation in NW (from B). (D) Heatmaps of response ratios (TW/NW or transgenic/WT) of cell wall remodelling genes from the intersections of C. Significant differences (*P* ≤ 0.05) are underlined. Data were from n = 3 for WT, n = 8 for A1dYB9, n = 5 for A1dEY(B15), or n = 2–3 for transgenic lines A1dEYB15-11 and A1dEY-5. AGP, arabinogalactan protein; FC, fold-change; PMEI, pectin methylesterase inhibitor; XTH, xyloglucan endotransglucosylase/hydrolase.

Vegetatively propagated plants were subjected to TW induction, a treatment known to increase both MT and MF abundance (Pilate *et al.*, 2004). X-ray µCT imaging analysis confirmed TW formation on the upper side of the inclined stem (Fig. 4A), which exhibited significantly higher density than the normal wood (NW; Fig. 4B). The expected lignocellulosic compositional shifts in TW were observed (Foston *et al.*, 2011), including significantly increased levels of glucose, arabinose, rhamnose, and galactose, and decreased xylose, mannose, and lignin (Fig. 4C-I), but there were no genotypic differences in either NW or TW. In agreement with the wood trait response, RNA-Seq analysis revealed large TW effects, but small genotypic differences in either NW or TW (Fig. 5). The small number of genes significantly up-regulated by tubulin perturbation overlapped substantially with those induced by TW (Fig. 5A-C), and, in effect, their response to TW was attenuated in the transgenics compared to WT (Fig. 5B,D). Many of these genes encode cell wall-modifying enzymes, such as pectin lyases (e.g. Biswal *et al.*, 2014), pectin acetylesterase (Gou *et al.*, 2012), cellulase (Lewis *et al.*, 2013), β-xylosidases (Goujon *et al.*, 2003), and α-expansins (Gray-Mitsumune *et al.*, 2008) (Fig. 5D, Supplementary Table S4). In fact, overexpression of pectin lyase Potri.003G175900 in transgenic *Populus* has been shown to alter cell wall pectin and xylan extractability without affecting the wood composition, similar to what was observed in this study. The RNA-Seq data thus provide molecular support for cell wall remodelling in the transgenics due to tubulin perturbation.

### Altered stomatal behaviour of transgenic leaves

**Fig. 6.**
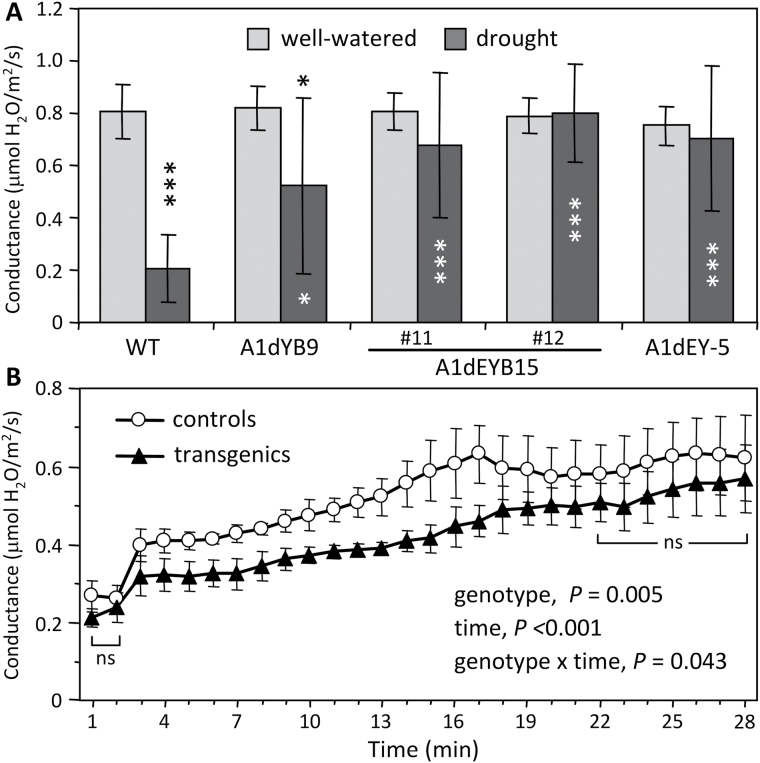
Stomatal responses of WT and transgenic leaves. (A) Stomatal conductance of mature leaves (LPI-15) under well-watered or drought conditions. Error bars are SD of n = 4–10 plants. Statistical significance of treatment effect was determined using the paired two-sample *t*-test (***P < 0.005; *P < 0.05). Asterisks above the bars are for treatment effects, and inside the bars are for transgenic effects. (B) Stomatal conductance of dark-acclimated mature leaves (LPI-10) in response to light. Error bars are SD of n = 5 control (WT and transgenic control) or transgenic (A1dYB9 and A1dEYB15) plants. Statistical significance was determined by repeated measures two-way ANOVA. ns, no significance based on the Student-Newman-Keuls *post-hoc* test.

Next, whether another MT-dependent process—the dynamic opening and closing of stomata—might also be affected in the transgenic plants was investigated. Putative changes in MT stability of the guard cells due to tubulin perturbation would be expected to alter stomatal conductance in response to external stimuli. Drought stress is known to induce stomatal closure, as shown by the significant decrease of stomatal conductance in WT (Fig. 6). However, transgenic plants showed delayed stomatal closure in response to drought (Fig. 6A, Supplementary Fig. S5). This slowed response led to the prediction that stomatal opening would also be delayed in the transgenics. Indeed, the rate of stomatal opening in response to light was slower in the transgenics than in the controls, whereas the steady-state stomatal conductance remained similar between genotypes under dark-acclimated or light-saturated conditions (Fig. 6B). These results imply that tubulin PTM perturbation in transgenic poplar altered some MT-dependent aspect of the stomatal response to environmental cues.

## Discussion

### Tubulin manipulation in transgenic *Populus* was facilitated by PTM mimics

In this study, transgenic *Populus* have been obtained with perturbed tubulin homeostasis. The overall transformation efficiency was very low, consistent with developmental defects and/or lethality frequently associated with tubulin manipulation (Weinstein and Solomon, 1990; Abe *et al.*, 2004). While *TUA–TUB* co-transformation to obtain balanced expression was deemed essential for viable transgenic tobacco (Anthony *et al.*, 1999), that strategy did not produce viable transgenic *Populus* in the present study. Instead, expressing a PTM mimic (dY or dEY) of *TUA1*, with or without *TUB*, was more effective for recovery of *Populus* transformants. Interestingly, the transcript levels of the *TUA* transgenes (*TUA1dY* or *TUA1dEY*) were several-fold higher than those of *TUB* transgenes in all transgenic lines examined, despite the use of a constitutive CaMV 35S promoter in all cases. This is reminiscent of previous findings that endogenous *TUA* transcripts are more abundant than *TUB* transcripts across a range of *Populus* tissues (Oakley *et al.*, 2007). The results suggest that both endogenous and introduced *TUA* and *TUB* genes are subject to post-transcriptional or co-translational regulation in *Populus*, presumably via auto-regulation or auto-feedback regulation as reported in animal systems, to maintain a proper ratio of the two subunits (Pachter *et al.*, 1987; Gonzalez-Garay and Cabral, 1996). The inherent *TUA*-biased transcript abundance in *Populus* also suggests that high levels of *TUB* transcripts, either endogenous or transgenic, may be detrimental for development, as reported in yeast (Weinstein and Solomon, 1990). This may explain the limited success of the tubulin co-transformation strategy in *Populus*.

Besides the *TUA* bias, expression of tubulin transgenes also exhibited a tissue-level bias, where higher transcript abundance was observed in the leaves than in xylem, opposite to the endogenous transcripts (Fig. 1). Although the CaMV 35S promoter exhibits slightly lower activity in xylem than leaves of *Populus* (Eriksson *et al.*, 2000; Babst *et al.*, 2014), it has been successfully used to overexpress genes in *Populus* xylem (Zhong *et al.*, 2011; Lin *et al.*, 2013). In support of its efficacy in xylem, transcripts of 35S-driven hygromycin phosphotransferase marker gene were detected at high levels (average FPKM 1011), on par with the xylem-abundant *TUB15* (FPKM 1023) and several lignin biosynthetic pathway genes in A1dYB9 (NW) xylem, based on RNA-Seq analysis (Supplementary Table S4). Thus, the unusually low levels of *TUA* and especially *TUB* transgene expression in xylem of all viable transgenic lines strongly suggests that higher levels might constrain recovery of developmentally normal *Populus* transformants. Further supporting this argument was the observation that most of the callus lines failed to advance during shoot regeneration, characteristically exhibiting defective vasculatures (Fig. S1). Together, these results underscore the complexity of tubulin regulation at multiple levels, which likely contributed to the difficulties in obtaining transgenic alteration of tubulin expression.

### Tubulin perturbation affected pectin–xylan networks in transgenic *Populus* wood

A subtle perturbation of the tubulin subunit pool was achieved by overexpressing PTM mimics of TUA in *Populus*. The perturbations were found to affect cell wall properties, but not in a manner predicted by the classic function of MTs in directing cellulose MF deposition. Results showed that pectin–xylan polysaccharide extractability and lignin S/G ratio, but not cellulose characteristics (content, MFA, or crystallinity), were sensitive to tubulin manipulation in the xylem. The absence of a clear effect on cellulose in the viable plants might be due to weak transgene expression in xylem as discussed above. It is plausible that strong transgenic effects might have compromised structural integrity as reported previously (Burk *et al.*, 2006; Ishida *et al.*, 2007), and contributed to regeneration failure in the present study. Altered matrix polysaccharide extractability could signify changes in structure, composition, and/or linkage. Because vascular structural integrity was maintained and cell wall carbohydrate levels did not change quantitatively in the transgenics, the differential extractability of cell wall glycans can be best explained by qualitative changes that alter the pectin–xylan networks.

Pectins are abundant in the primary cell wall and middle lamella, and their deposition precedes that of cellulose, hemicelluloses, and lignin (Keegstra, 2010). At the onset of secondary cell wall formation, the primary cell wall is pushed outwards into the middle lamella and cell corners between adjoining cells (Jarvis *et al.*, 2003) where lignin (typically G lignin) deposition first occurs (Boerjan *et al.*, 2003; Tobimatsu *et al.*, 2013). The preferential increase of G lignin, and the enhanced extractability of multiple pectic (arabinan, AG, RG-I, and de-esterified HG) epitopes in the lignin-bound cell wall fractions of transgenic wood are both consistent with a close association between early-deposited pectin and lignin polymers in the middle lamella and cell corners. This association likely involved xylan, because several xylan epitopes also exhibited increased extractability in the more tightly bound cell wall extracts of the transgenics. In support of this view, a recent study showed that the *Arabidopsis irregular xylem 8* (*irx8*) mutant deficient in xylan and HG owing to a mutation in galacturonosyltransferase is compromised in lignin biosynthesis (Hao *et al.*, 2014). Specifically, defective matrix polysaccharide deposition led to reduced accrual and altered extractability of G lignin in the *irx8* mutant (Hao *et al.*, 2014). These and observations from the current study strongly support a close association of pectin, xylan, and lignin during early stage of cell wall biogenesis.

Pectins and other matrix polysaccharides are synthesized in the Golgi and transported to the plasma membrane for deposition into the cell wall (Keegstra, 2010), a process that implicates MT-mediated vesicle trafficking (Crowell *et al.*, 2009; Hamada *et al.*, 2012). A role for MTs in pectin deposition has been inferred based on immunofluorescence microscopy and pharmacological studies. Pectin vesicles were observed in close association with cortical MTs near the mucilage secretion pockets during *Arabidopsis* seed coat development (McFarlane *et al.*, 2008). Oryzalin treatment was shown to affect not only cellulose but also pectin deposition in unicellular green alga (Domozych *et al.*, 2014). Two recent studies showed that *Arabidopsis* mutants defective in kinesin motor proteins exhibited abnormal Golgi morphology, with altered pectin secretions and accumulation of arabinose-containing carbohydrates in the cell wall (Kong *et al.*, 2015; Zhu *et al.*, 2015). It is worth noting that tubulin C-termini are exposed on the outer surface of MTs where their interactions with a host of MT-associated proteins (MAPs) modulate MT dynamics (Janke and Bulinski, 2011). It is conceivable that tubulin PTM perturbation interfered with MT-mediated vesicle trafficking and affected pectin polysaccharide deposition and its integration with xylan and lignin polymers. The involvement of spatiotemporally regulated MAPs (Sedbrook and Kaloriti, 2008; Gardiner, 2013) may underscore the conditional nature of the phenotype affecting mostly early cell wall deposits.

### Stomatal behaviour was sensitive to tubulin perturbation in transgenic *Populus*

Tubulin perturbation delayed drought-induced stomatal closure as well as light-induced stomatal opening without changing steady-state stomatal behaviour in transgenic *Populus*. A dynamic relationship between MTs and stomatal function has been unequivocally demonstrated using immunofluorescence microscopy or live cell imaging coupled with pharmacological treatments (Fukuda *et al.*, 1998; Eisinger *et al.*, 2012). The MT-depolymerizing drug oryzalin prevents stomatal opening, whereas the MT-stabilizing drug taxol impedes stomatal closure (Fukuda *et al.*, 1998; Eisinger *et al.*, 2012). Thus, the observed delay in stomatal closure in drought-stressed leaves may be attributed to more stable MT arrays due to ectopic expression of tubulin PTM mimics. This is consistent with the frequent association of dY- and dEY-TUA isoforms with stable MTs in animal systems (Peris *et al.*, 2009; Janke and Kneussel, 2010). On the other hand, light-induced stomata opening requires polymerization of tubulins (Eisinger *et al.*, 2012). The delayed response observed in transgenic *Populus* raises the possibility that MT polymerization was slowed owing to altered tubulin pool composition. In support of this idea, dY-TUA has been shown to assemble more slowly than the tyrosinated counterpart in taxol-promoted *in vitro* polymerization (Banerjee and Kasmala, 1998). In addition, the delayed stomatal responses may hint at altered guard cell wall properties as a result of tubulin perturbation. Guard cell wall chemistry is not well understood, but studies based on immunolocalization and Fourier transform infrared spectroscopy all point to pectins as the major component imparting strength and flexibility necessary for dynamic change of cell shape during stomatal opening and closure (Majewska-Sawka *et al.*, 2002; Jones *et al.*, 2003; Jones *et al.*, 2005). Based on this, and on the glycome profiling results showing that the pectin–xylem networks were sensitive to tubulin perturbation, one may speculate that the transgenic effect on pectins also occurred in guard cells to impact stomatal movement.

### Empirical evidence against an active tubulin tyrosination–detyrosination cycle in *Populus*

The transgenic phenotypes of plants expressing either *TUA1dY* or *TUA1dEY* PTM mimics were similar, differing only in degree. The comparatively strong phenotypes of A1dEYB15/A1dEY relative to A1dYB9 plants were consistent with higher TUA1dEY than TUA1dY signals at both RNA and protein levels in the respective lines (Figs. 1 and 2). The possibility was considered that the phenotypic variation was due to differing capacities of the two PTM mimics to participate in the tyrosination–detyrosination cycle (i.e. TUA1dY could be retyrosinated back to TUA1 or converted to TUA1dEY, but TUA1dEY could not be modified further), but several lines of evidence argue against this. First, despite the wealth of knowledge about tubulin tyrosination/detyrosination in animal systems, support for such a cycle in plants has only been inferred from antibody-based detection (Smertenko *et al.*, 1997; Wang *et al.*, 2004). In fact, the dY- and dEY-TUA1 isoforms that might be indicative of a tyrosination–detyrosination cycle were essentially undetected in WT xylem by western blot and proteomics analyses in the present study. Second, no plant orthologues of the mammalian tubulin tyrosine ligase (Ersfeld *et al.*, 1993) involved in dY-to-Y retyrosination have been identified (Oakley *et al.*, 2007). The mouse cytosolic carboxypeptidase 1 reported to catalyse the irreversible dY-to-dEY conversion (Berezniuk *et al.*, 2012) also lacks any apparent orthologue in plants (Phytozome v10.2), and indeed, no evidence of dY-to-dEY conversion in the A1dYB9 line was found. Together, these data seem to suggest that the tyrosination–detyrosination cycle, evolutionarily conserved from animals to parasitic protozoa (Xiao *et al.*, 2009), is absent in plants. How then did the dY/dEY PTM mimics of TUA elicit an effect in transgenic *Populus*? Tubulin C-terminal PTMs have been shown to affect MT assembly or interactions with MAPs (Konishi and Setou, 2009; Peris *et al.*, 2009; Sirajuddin *et al.*, 2014). Given that TUA1dY/dEY isoforms are normally absent in *Populus*, their ectopic expression in the transgenics may represent ‘foreign’ tubulin codes that can interfere with MAP interactions. Interestingly, among the suite of annotated plant MAPs (Hamada *et al.*, 2013), only two putative *Populus* orthologues showed significantly altered (reduced) expression in all transgenic plants (Supplementary Table S4). The two genes (Potri.006G018000 and Potri.016G006900) are genome duplicates and orthologous to *Arabidopsis* AtMAP70-5, which is known to regulate secondary cell wall polymer organization (Pesquet *et al.*, 2010). Although further research is needed to elucidate the interplay between MAP70-5 and cell wall polysaccharide organization in *Populus*, the data support the idea that tubulin PTM perturbation can modulate MAPs to affect MT-dependent processes.

A remaining question is whether the observed phenotypes were due to tubulin PTM perturbation specifically, or to altered tubulin abundance/composition in general, because transgenic lines expressing native tubulin genes were not obtained in the present investigation. Given the inherently disproportionate *TUA* and *TUB* transcript abundance in *Populus*, manipulating single tubulin expression should be feasible to address this question.

## Supplementary data

Supplementary data are available at *JXB* online.

Supplementary Fig. S1. Abnormal vascular development was observed in nonviable transgenic lines.

Supplementary Fig. S2. PCR analysis of wild-type and transgenic plants using gene-specific primers.

Supplementary Fig. S3. Stem cross sections of wild-type and transgenic plants.

Supplementary Fig. S4. Total sugars and glycosyl composition of sequential cell wall extracts from wild-type and transgenic plants.

Supplementary Fig. S5. Photosynthetic responses of wild-type and transgenic leaves from replicate drought experiments.

Supplementary Table S1. Primer information

Supplementary Table S2. Summary of transformation response from multiple trials.

Supplementary Table S3. ELISA hybridization signal intensities from glycome profiling of wood tissues.

Supplementary Table S4. Gene expression analysis by RNA-Seq.

Supplementary Data

## Abbreviations:

AG: arabinogalactan
CaMV: cauliflower mosaic virus
dEY: non-tyrosinatable
dY: detyrosinated or detyrosination
G lignin: guaiacyl lignin
HG: homogalacturonan
LPI: leaf plastochron index
mAb: monoclonal antibody
MALDI: matrix-assisted laser desorption/ionization
MAP: MT-associated protein
MF: microfibril
MFA: microfibril angle
MT: microtubule
NW: normal wood
PTM: post-translational modification
RG: rhamnogalacturonan
S lignin: syringyl lignin
TOF: time-of-flight
TUA: α-tubulin
TUB: β-tubulin
TW: tension wood
μCT: micro computed tomography
WT: wild type.
