# Supplementary material for: Tubulin perturbation leads to unexpected cell wall modifications and affects stomatal behaviour in Populus
Source: J Exp Bot. 2015 Aug 5;66(20):6507–18. doi: 10.1093/jxb/erv383 (PMC4588895; doi:10.1093/jxb/erv383)
Supplement: Supplementary Data [file supp_66_20_6507__index.html]

Tubulin perturbation leads to unexpected cell wall modifications and affects stomatal behaviour in Populus — Tubulin perturbation leads to unexpected cell wall modifications and affects stomatal behaviour in Populus — Supplementary Data 

# Tubulin perturbation leads to unexpected cell wall modifications and affects stomatal behaviour in *Populus*

## Supplementary Data

Data files

- Supplementary Data - Supplementary Data
- Supplementary Data - Supplementary Data
- Supplementary Data - Supplementary Data
