# Supplementary material for: Tubulin perturbation leads to unexpected cell wall modifications and affects stomatal behaviour in Populus
Source: J Exp Bot. 2015 Aug 5;66(20):6507–18. doi: 10.1093/jxb/erv383 (PMC4588895; doi:10.1093/jxb/erv383)
Supplement: Supplementary Data [file supp_erv383_jexbot151712_file001.pdf]

Supplemental Table S1. Primer information

Gene	Forward and reverse primers	Purpose
TUA1	F: GCTCTAGATGAGAGAGTGCATTTCGATTCA<br>R: TCCCCCGGGTCAGTACTCATCTCCTTCAT	Cloning
TUA1dY	F: GCTCTAGATGAGAGAGTGCATTTCGATTCA<br>R: ACGCGTCGACTCACTCATCTCCTTCATCACCAT	Cloning
TUA1dEY	F: GCTCTAGATGAGAGAGTGCATTTCGATTCA<br>R: ACGCGTCGACTCAATCTCCTTCATCACCATCCT	Cloning
TUB9	F: GCTCTAGATGAGAGAAATCCTTCATG<br>R: TCCCCCGGGTTAGTTCTCCATAGGCTCTT	Cloning
TUB15	F: GCTCTAGATGAGAGAAATCCTTCACATTC<br>R: TCCCCCGGGCTAGGCAGCCTCTTCCTCCT	Cloning
TUA1dY/TUA1dEY transgenes	F: GCTCTAGATGAGAGAGTGCATTTCGATTCA<br>R: ATCGCAAGACCGGCAACAGG	genomic PCR
TUB9 transgene	F: GCTCTAGATGAGAGAAATCCTTCATG<br>R: ATCGCAAGACCGGCAACAGG	genomic PCR
TUB15 transgene	F: GCTCTAGATGAGAGAAATCCTTCACATTC<br>R: ATCGCAAGACCGGCAACAGG	genomic PCR
Glycosyltransferase (housekeeping)	F: TTGGGCTTGAGCAAAGTGG<br>R: AGATCCACCCTCCAACAGTG	genomic PCR
HPT	F: GAGGGCGAAGAATCTCGTGC<br>R: GATGTTGGCGACCTCGTATTG	genomic PCR
NPT	F: GAACAAGATGGATTGCACGC<br>R: GAAGAACTCGTCAAGAAGGC	genomic PCR
TUA1 endogene	F: CGATGGAGAGGATGGTGATGAAGGA<br>R: CACGTACCAACAGACATGGTCTAAGC	qRT-PCR
TUB9 endogene	F: CAGTCATGTTTAGGAGAAARGCGT<br>R: CAACTCATCCTTGAAGCCATRGCC	qRT-PCR
TUB15 endogene	F: ACAGCTATGTTCAGGAGGAAGGCT<br>R: TCACACCAGCCACATGGCTTAMTA	qRT-PCR
TUA1dY/TUA1dEY transgenes	F: CGATGGAGAGGATGGTGATGAAGGA<br>R: TAATCATCGCAAGACCGGCAACAG	qRT-PCR
TUB9 transgene	F: CAGTCATGTTTAGGAGAAARGCGT<br>R: TAATCATCGCAAGACCGGCAACAG	qRT-PCR
TUB15 transgene	F: ACAGCTATGTTCAGGAGGAAGGCT<br>R: TAATCATCGCAAGACCGGCAACAG	qRT-PCR
elongation factor 1-β (housekpeeing)	F: GACCTKGTATCAGTGGATTCCCTC<br>R: GAACAGAGGCACAAGATTACCAGG	qRT-PCR
actin (housekpeeing)	F: GCGGTGATGGTGAGTTCTTTCT<br>R: ATCGAGAGGGAGGACCATTACAGT	qRT-PCR
actin-related protein (housekpeeing)	F: ACTGTGAGGAGATGCAGAAACGCA<br>R: GCTGTGTCACGGGCATTCAATGYT	qRT-PCR

Supplemental Table S2. Summary of transformation response from multiple trials

**Construct pair**	**explant**	**callus**[a]	**shoot**[a]	**root**[a]
TUA1+TUB9	106	8	1	0
TUA1+TUB15	100	6	1	0
TUA5+TUB9	98	3	0	0
TUA5+TUB15	100	6	1	0
TUA1dY+TUB9 (A1dYB9)	114	11	3	1
TUA1dY+TUB15	90	10	2	0
TUA1dEY+TUB9 (A1dEYB9)	118	15	2	2
TUA1dEY+TUB15 (A1dEYB15)	103	17	11	4[c]
Vector control [b]	42	> 15	> 5	5

[a]Numbers of putative independent events are shown.

[b]Only subsets of events were advanced through the regeneration process.

[c]One event was later confirmed to contain only the TUA1dEY transgene.

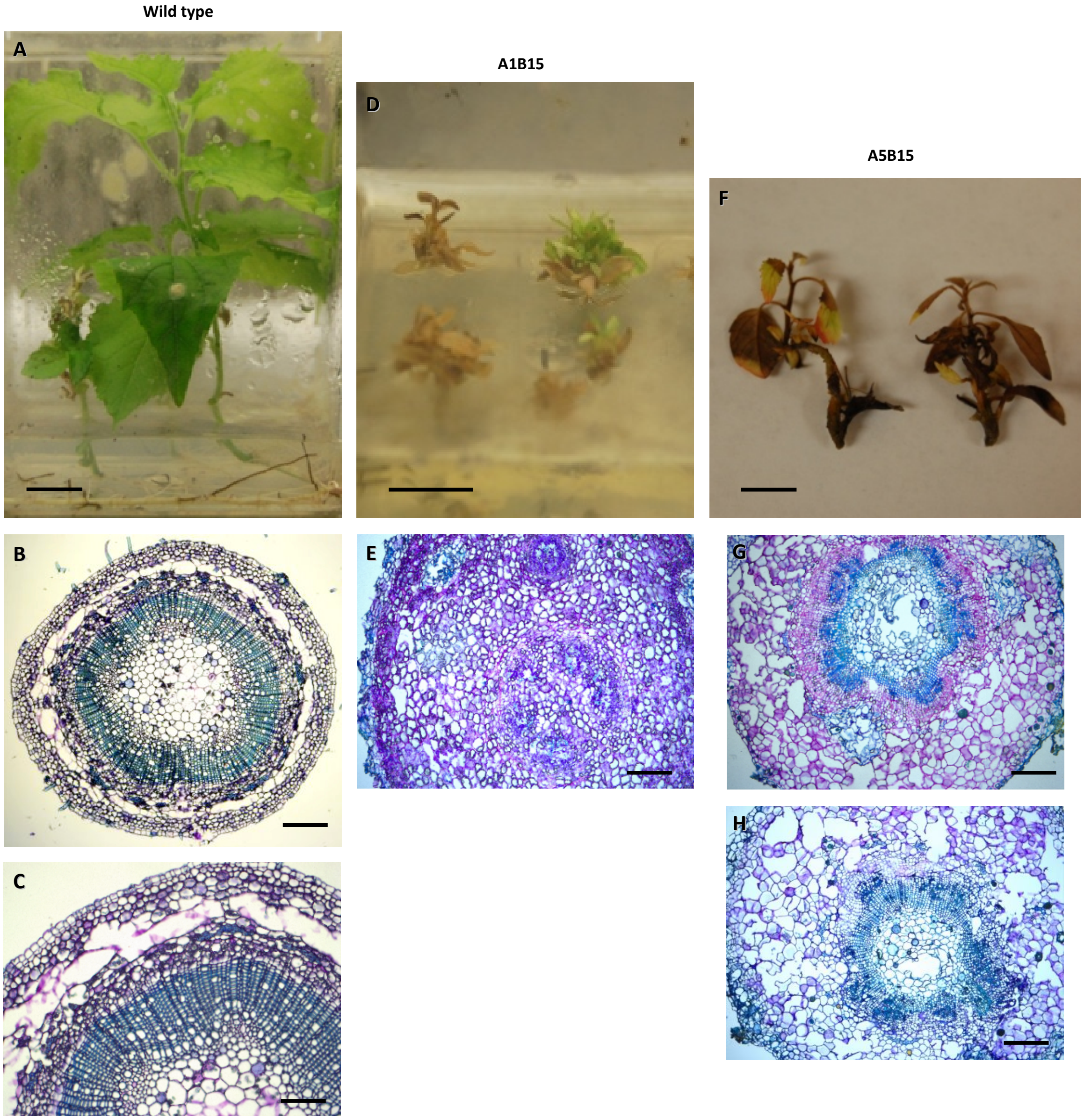

Supplemental Fig. S1. Abnormal vascular development was observed in nonviable transgenic lines. A-C, Wild type; D-E, transformants derived from co-transformation of *TUA1* and *TUB15 (*A1B15); F-H, transformants derived from co-transformation of *TUA5* and *TUB15* (A5B15). Scale bar = 1 cm (A, D, F), 500 µm (B) or 125 µm (C, E, G, H).

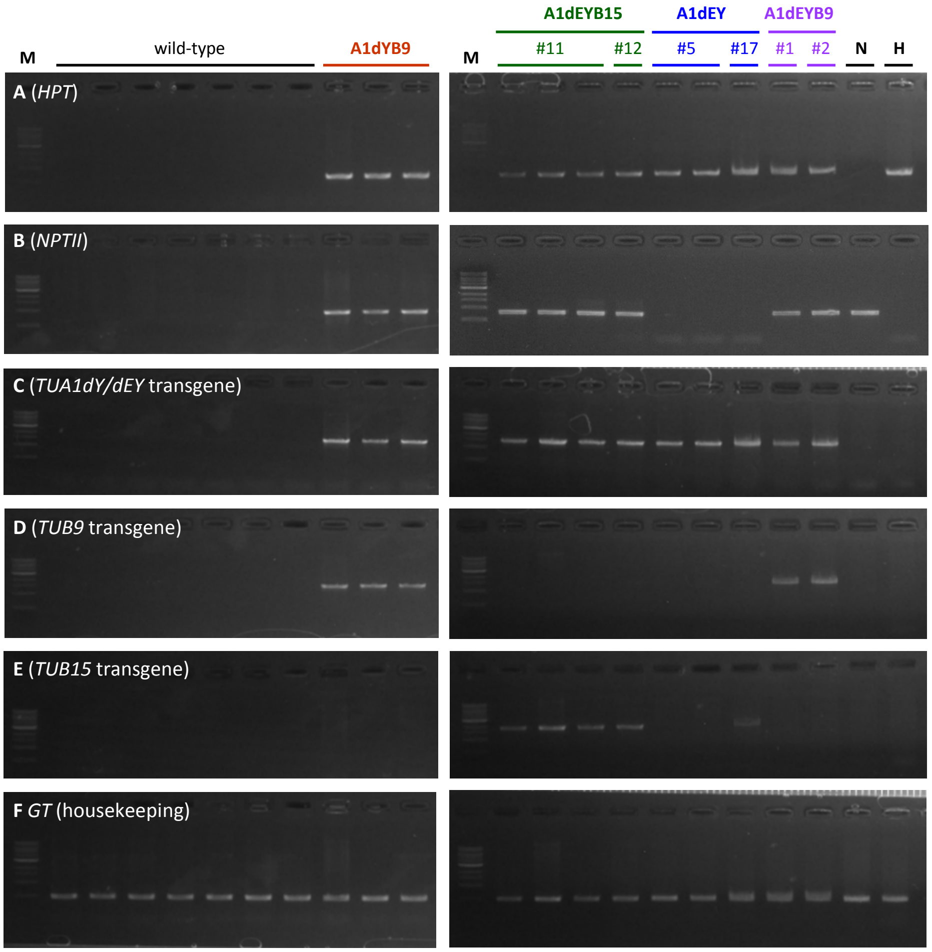

Supplemental Fig. S2. PCR analysis of wild-type and transgenic plants using gene-specific primers. (**A**) Selectable marker hygromycin phospho-transferase (*HPT*) gene from the *TUA* constructs. (**B**) Selectable marker neomycin phosphotransferase II (*NPTII*) gene from the *TUB* constructs. (**C**) *TUA1/dY/dEY* transgene. (**D**) *TUB9* transgene. (**E**) *TUB15* transgene. (**F**) A single-copy glycosyltransferase (*GT*) gene Potri.016G014500 as housekeeping control. A1dY, TUA1dY; A1dEY, TUA1dEY; B9, TUB9; B15, TUB15; M, molecular weight markers; N and H, vector controls with the NPTII or HPT construct, respectively. The A1dEY #5 and #17 lines was originally obtained from transformation with the A1dEYB15 combination, but found to harbor only the *TUA* and *HPT* transgenes.

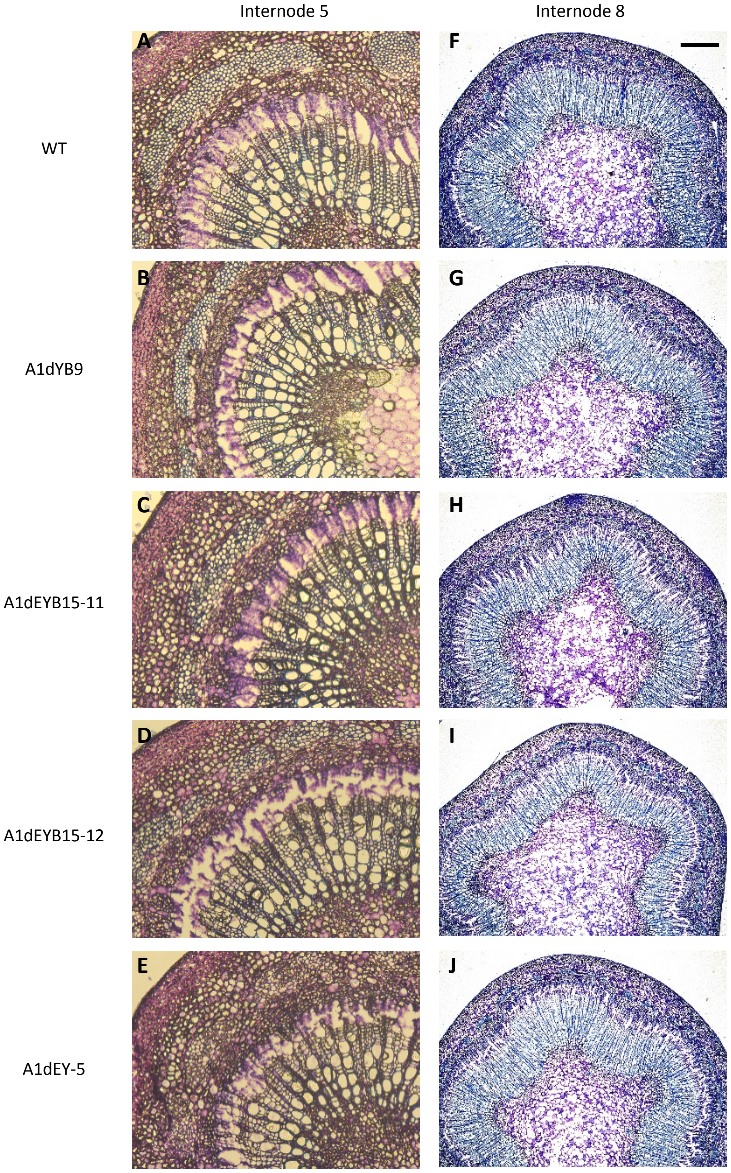

Supplemental Fig. S3. Stem cross sections of wild-type and transgenic plants. Paraffin sections (10 μm) were stained with toluidine blue and visualized under a light microscope. Scale bar = 125 μm (A-E) or 500 μm (F-J).

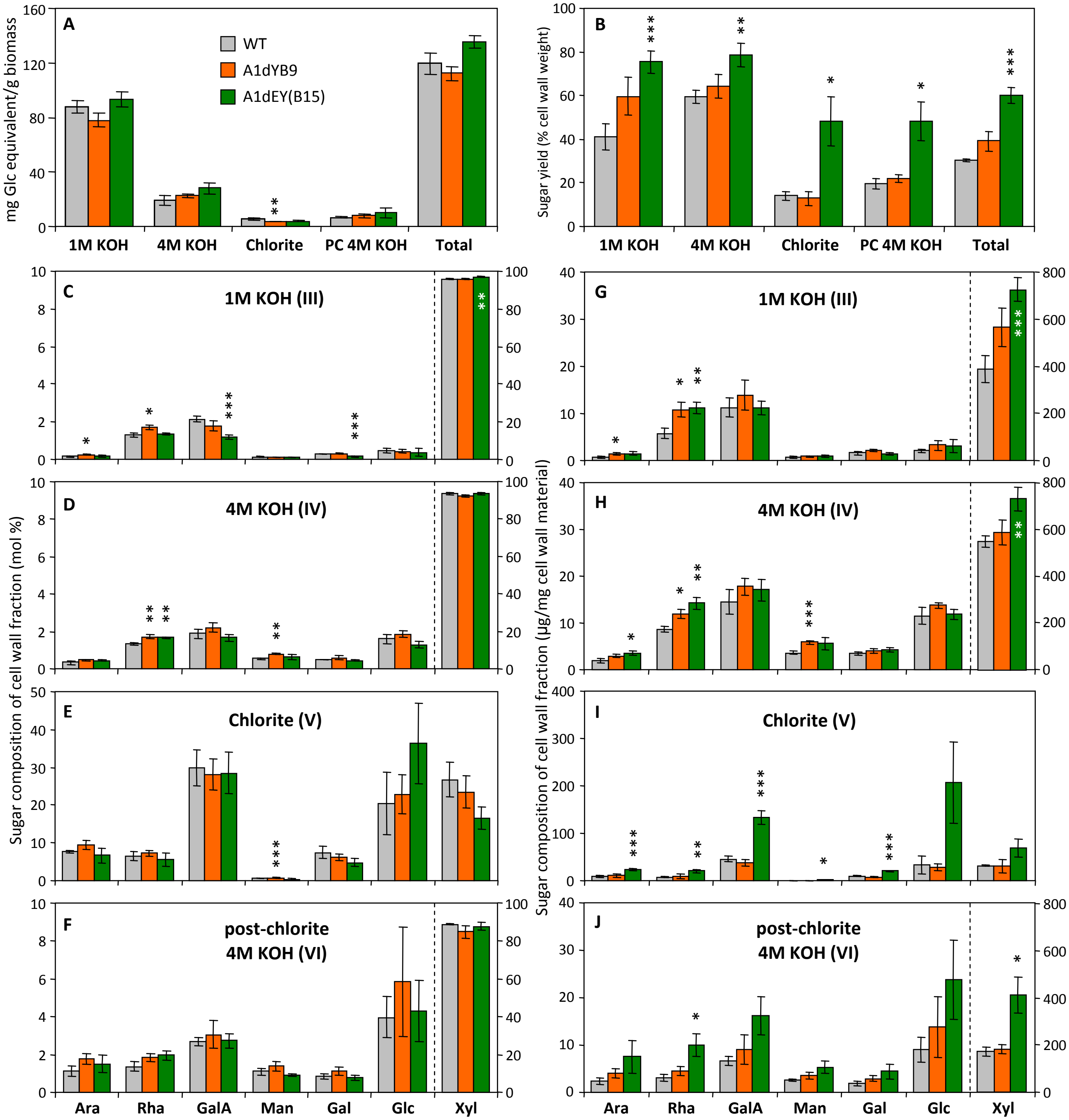

Supplemental Fig. S4. Total sugars and glycosyl composition of sequential cell wall extracts of wild-type and transgenic plants. (**A**) Total sugars in each cell wall fraction as measured by the phenol-sulfuric acid method. (**B**) Total sugar yield in each cell wall fraction recovered by methanolysis. (**C-J**) Sugar composition expressed as mol % (C-F) or as µg sugar/g cell wall material (G-J) in each fraction. Data are mean ± SE of n=3-9 in (A) or n= 3-5 in (B-J). Refer to the right axis for Xyl in C, D, F, G, H and J. Statistical significance was determined by two-sample *t*-test (*** $P \leq 0.01$; ** $P < 0.05$; * $P < 0.1$).

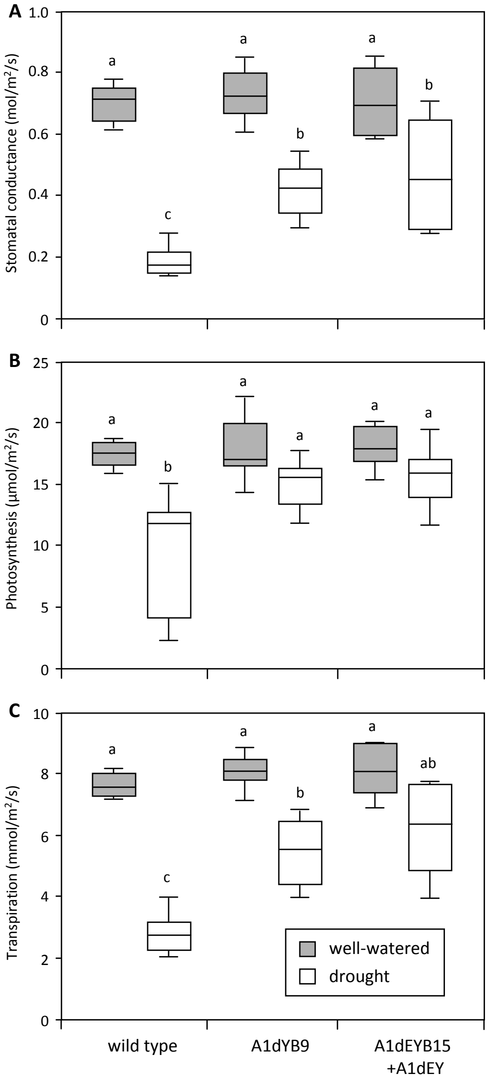

Supplemental Fig. S5. Photosynthetic responses of wild-type and transgenic leaves from replicate drought experiments. (**A**) stomatal conductance, (**B**) net photosynthesis, and (**C**) transpiration rates of LPI-15 under well-watered or drought conditions. Statistical significance was determined by one-way ANOVA (n= 3 for WT and 6-7 for the transgenics).
